# Influences of Multilayer Graphene and Boron Decoration on the Structure and Combustion Heat of Al_3_Mg_2_ Alloy

**DOI:** 10.3390/nano10102013

**Published:** 2020-10-13

**Authors:** Jun Dong, Weili Wang, Xiaofeng Wang, Shaojun Qiu, Maohua Du, Bo Tan, Yanjing Yang, Taizhong Huang

**Affiliations:** 1 College of Ordnance Engineering, Naval University of Engineering, Wuhan 430033, China; J.Dong204@mail.cri.cn (J.D.); MH_Du@nue.edu.cn (M.D.); N_Tan@nue.edu.cn (B.T.); 2 Xi’an Modern Chemistry Research Institute, Xi’an 710065, China; qiu_shaojun@mail.cri.cn (S.Q.); mse_yyj@mail.cri.cn (Y.Y.); 3 School of Chemistry and Chemical Engineering, University of Jinan, Jinan 250022, China

**Keywords:** Al_3_Mg_2_ alloy, multilayer graphene, boron, combustion heat

## Abstract

To improve the engine-driven performance of propellants, high-energy alloys such as Al and Mg are usually adopted as annexing agents. However, there is still room for improvement in the potential full utilization of alloy energy. In this study, we investigated how to improve combustion efficiency by decorating Al_3_Mg_2_ alloy with multilayer graphene and amorphous boron. Scanning electron microscopy and Raman tests showed that decorating with multilayer graphene and amorphous boron promoted the dispersion of Al_3_Mg_2_ alloy. The results showed that decorating with 1% boron and 2% multilayer graphene improved the combustion heat of Al_3_Mg_2_ alloy to 32.8 and 30.5 MJ/kg, respectively. The coexistence of two phases improved the combustion efficiency of the matrix Al_3_Mg_2_ alloy.

## 1. Introduction

Energetic materials have been widely used in propellants. With increased demand for high-energy-density materials, more metals, such as Al, Mg, and Ti, have been investigated [1,2]. Al-based materials are still the most popular research topic in the field of energetic materials, despite having been used for dozens of years [3]. Recently, Pang et al. reported on the combustion behavior of AP/HTPB/Al composite propellants containing a hydroborate iron compound [4]. The results showed that the combustion characteristics were improved by the introduction of 5% hydroborate iron. The combustion of Al particles is usually inhibited by the oxide layer of Al_2_O_3_ on the surface. Different researchers have studied how to remove aluminum-based oxide on the surface. One study showed that a CuF_2_ solution can simultaneously remove the Al_2_O_3_ layer on Al particles and form the Cu–Al nanocomposite [5]. The maximum heat release of the Cu–Al energetic nanocomposites with 5% Cu content reaches 24.7 kJ g^−1^, which is approximately 1.8 times that of raw Al. 

In addition to the high purity Al powder, Al-based alloys such as Al–Mg binary alloys have been investigated as additive materials for propellants. To improve the combustion performance of Al–Mg-based micron particles, zirconium particles are modulated to the matrix [6]. The results clearly showed that compared to commercial Al powder, the combustion efficiency of the composite materials increases from 30% to 80–90%. Al-based materials have been developed to enhance the specific impulse of solid composite propellants. For example, metal compounds such as Fe(IO_3_)_3_ and Cu(IO_3_)_2_ have been adopted to modify the Al powder [7]. The results showed that preparing a core-shell structure to provide an interfacial reaction with gas production release was important for the size reduction of agglomerated Al in µ-Al@M(IO_3_)_x_ for the combustion of solid composite propellants. To enhance the combustion performance of aluminum, ultra-fine iron and amorphous boron additives have been added to Al-based propellants [8]. When Alex is replaced by 2 wt% of boron, the burning rate is practically unchanged compared that of basic propellant with Alex. However, the agglomeration of combustion products is significantly enhanced. The content of agglomerated particles increased by 1.8–2.2 times. 

The aluminothermic reactions of Al/CuO and Al/MnO_2_ have also been employed to fully use the Al-based materials in propellants [9,10,11,12]. The results showed that thermites can reduce agglomeration and decrease diffusion distance more than common mechanically mixed materials. The ignition and combustion properties of thermites can be characterized with a constant-volume vessel, bomb calorimetry, and differential scanning calorimetry tests. Al–Mg-based alloys have also been adopted in propellants [13]. The combustion of Al–Mg alloy powders can be improved with a coating of fluoropolymer [14]. Based on the experimental pressure traces, it was estimated that the fluoropolymer-coated alloys can achieve higher burning velocity. Despite many studies reporting that the combustion of Al–Mg-based alloys can be modulated, a method to improve the combustion performances of Al–Mg-based alloys has yet to be constructed. Applying Al–Mg-based alloys as a matrix to develop novel materials for propellants has therefore attracted great attention in recent years.

In addition to Al-based materials, boron-based materials have also been widely investigated by researchers. Boron is usually used in amorphous states [15]. The combustion activity of amorphous boron can be modulated with the high-energy ball milling and combustion synthesis method. To fully use the potential performance of boron nanoparticles, they are embedded in paraffin wax [16]. Zhou et al. studied the combustion properties of boron particles [17] and found that the presence of fluorine significantly decreases the overall burn time in kinetically controlled burning systems but increases the time in diffusive controlled systems. To improve the combustion performances of B and other materials, carbon nanotubes (CNTs) and graphene have also been used [18,19]. The onset reaction temperature, ignition delay time, and flame structure of poly tetra fluoroethylene (PTFE)/Al with CNTs and graphene change significantly.

In this paper, we report the structure and combustion heat of multilayer graphene and boron particles modulated with Al_3_Mg_2_ alloys. The results show that the Al_3_Mg_2_ particles can be encapsulated by multilayer graphene, which promotes the combustion of the alloys and enhances combustion heat. Boron particles can also be mounted on the surface to decorate the Al_3_Mg_2_ particles and enhance the combustion heat of the Al_3_Mg_2_ alloy.

## 2. Materials and Methods

### 2.1. Materials

The Al and Mg powders and amorphous boron were obtained from the Aladdin Chemical Reagent Co., Ltd. (Shanghai, China). The diameters of the Al and Mg were distributed in the range of 3~5 µm. The particles of amorphous boron were about 100 nm in diameter. The multilayer graphene was obtained through improved Hummers’ methods in our laboratory [20]. It was difficult to find oxygen in the prepared graphene, which was reduced at 1000 °C for 2 h in a high-purity hydrogen atmosphere with a flow rate of 20 mL/min.

### 2.2. Synthesis of Materials

The Al_3_Mg_2_ alloy was prepared by induction melting in a water-cooled copper crucible (Crucible 2000, Nanjing University, Nanjing, China) in an argon atmosphere. The ingot was re-melted three times to ensure the homogeneity of the alloy. Then the alloy was mechanically milled in an argon atmosphere by a high energy grinding machine (GTB-200 Changsha Tianhong Co. Ltd., Changsha China) and sieved to obtain a grey powder with particle diameter less than 50 nm. The amorphous boron-decorated and multilayer graphene-decorated Al_3_Mg_2_ alloys were also obtained by high-energy ball milling methods in an argon atmosphere with a weight ratio of ball to materials of 10:1. The ground materials were heat to 600 °C at a rate of 5 °C/min in a cylinder oven (Hefei Kejing Co. Ltd., Hefei, China) and kept 1 h in an argon atmosphere. Then the heated materials were cooled down to ambient temperature naturally.

### 2.3. Apparatus and Measurements

Thermogravimetric (TG) analysis and synchronously differential scanning calorimeter (DSC) tests were carried out using a TA SDT 2960 thermoanalyzer (New Castle, DE, USA) with a heating rate of 5 °C min^−1^ in an argon atmosphere. Nitrogen adsorption-desorption isotherm measurements were performed using a Micromeritics ASAP 2020 analyzer (Norcross, GA, USA) to investigate the specific surface area. Before analysis, the multilayer graphene was pretreated by degassing at 200 °C for 8 h to remove any adsorbed species. X-ray powder diffraction (XRD) tests were conducted using a Bruke D8 focus instrument (Karlsruhe, Germany) with graphite-filtered Cu-Kα radiation. The scanning speed was 5 degrees per minute, with a step of 0.02 degrees from 10 to 80 degrees. SEM tests were conducted using a Hitachi (S-4800) scanning electron microscope (SEM) (Toyota, Japan) equipped with an energy-dispersive spectroscopy analyzer with charges of 15 kV and 10 pA. The combustion heat was examined with oxygen bomb equipment with an oxygen pressure of 10 MPa. The combustion heat tests were conducted 5 times and the average value of the tests was provided to eliminate the differences caused by the test system.

## 3. Results

### 3.1. Nitrogen Adsorption-Desorption Isotherm Test with Multilayer Graphene

**Figure 1:**
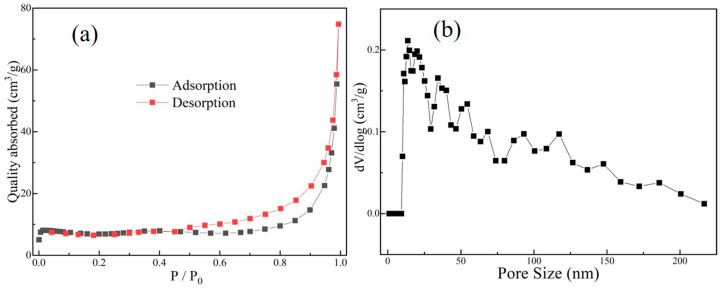
(**a**) The nitrogen adsorption-desorption isotherm curve and (**b**) the corresponding pore size distribution of multilayer graphene.

Figure 1 shows the nitrogen adsorption-desorption isotherm test results from the multilayer graphene. The N_2_ adsorption-desorption curve is clearly a type III curve [19]. The tests showed that the multilayer graphene had a relatively large Brunauer–Emmett–Teller (BET) specific surface area of 83.67 m^2^g^−1^. The pore size distribution of the multilayer graphene was also obtained and is displayed in Figure 1b, which clearly shows that there was a wide pore size distribution in the range of 30~200 nm, that is, in the range of mesoporous and large porous [20,21]. The wide distribution range of the pore size provided enough channels for the Al_3_Mg_2_ particles to be loaded into the interlayer space of the multilayer graphene.

### 3.2. Structure Tests with the Al_3_Mg_2_ Alloy

**Figure 2:**
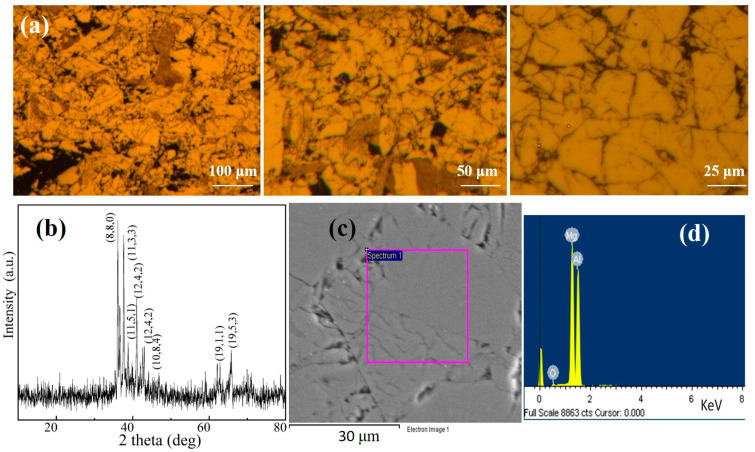
(**a**) The metallography of the Al_3_Mg_2_ alloy with different magnification scales, (**b**) XRD patterns of the Al_3_Mg_2_ alloy, and (**c**,**d**) scanning electron microscopy and corresponding energy dispersive spectra of the Al_3_Mg_2_ alloy.

The structure of the Al_3_Mg_2_ alloy was first examined via the metallography method. The results are shown in Figure 2. 

Figure 2a shows the metallography of the Al_3_Mg_2_ alloy with different magnifications. The grain boundary of the alloy can be clearly observed. There were no obvious impurities in the alloy. To further investigate the structure of the alloy, an X-ray diffraction (XRD) test was also conducted on the alloy, the results of which are shown in Figure 2b. Based on the XRD tests, the corresponding JCPDS number of the Al_3_Mg_2_ alloy was easily indexed as 29-0048, which clarified that the alloy had a cubic structure and a space group of Fd-3m(227). A similar structure was observed by other researchers [22]. Figure 2b also clearly shows that almost all the peaks could be indexed, which means that the alloy had a perfect crystallinity structure. Figure 2c,d show the scanning electron microscopy (SEM) and corresponding energy dispersive spectra (EDS) of the Al_3_Mg_2_ alloy, respectively. There were no impurities on the crystal particle, which is consistent with the results from the XRD tests. The EDS in Figure 2d showed that the oxygen signal was observed in addition to Al and Mg, which should be absorbed from the environment when preparing the samples for testing. Similar phenomena were also observed in studies of vanadium carbide-based alloys [23].

### 3.3. TG–DSC Tests with Amorphous Boron

**Figure 3:**
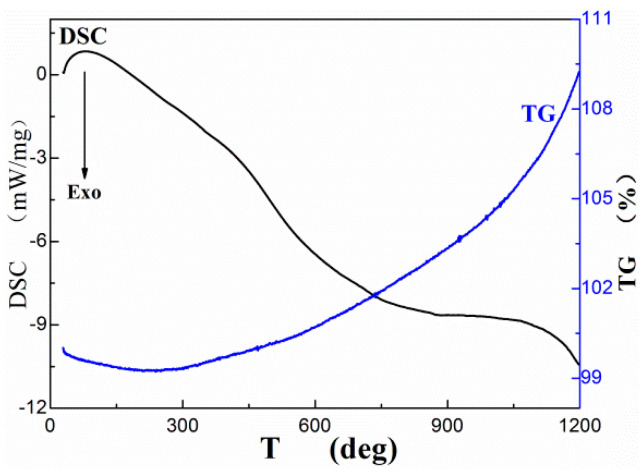
The TG and DSC test results of amorphous boron.

Thermogravimetric (TG) analysis and differential scanning calorimeter (DSC) tests were conducted on amorphous boron. The results are shown in Figure 3. The TG test results clearly revealed that the weight of the boron slightly decreased with an increase in temperature to 280 °C. This can be attributed to the evaporation of easily volatilized materials on the surface, such as absorbed H_2_O and certain gases. However, the weight of the sample slightly increased with the increase in temperature. This can be attributed to the oxidation of boron by trace oxygen in the argon carrier gas.

(1) $$B+O_{2}\to B_{2}O_{3}$$

Correspondingly, the DSC curve increased slightly at first due to the evaporation of materials on the surface of the boron. The curve then decreased with an increase in temperature, which can be attributed to the exothermal reaction caused by the oxidation of trace oxygen in the argon carrier gas.

### 3.4. Raman Tests with Multilayer Graphene

**Figure 4:**
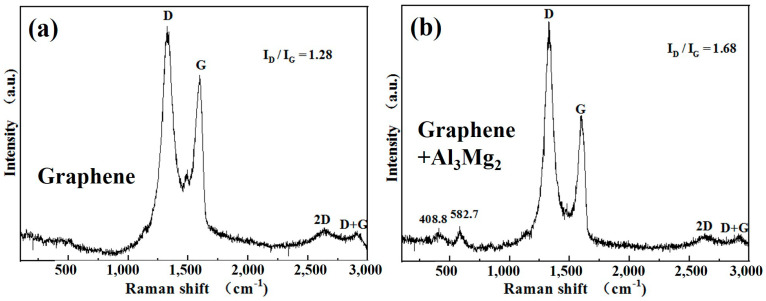
Raman tests on (**a**) multilayer graphene and (**b**) the multilayer graphene-decorated Al_3_Mg_2_ alloy.

To investigate the mutual interaction between the Al_3_Mg_2_ alloy and multilayer graphene, Raman tests were conducted on the multilayer graphene and the composite of the multilayer graphene-decorated Al_3_Mg_2_ alloy. The results are shown in Figure 4.

Figure 4a shows the Raman spectra of multilayer graphene, with the typical disorder (D) and graphitic (G) peaks that usually correspond to the defect structure and graphite structure [24]. The typical D and G bands are caused by the A_1g_ zone-edge phonon mode induced by the disorder of finite crystal-sized effects and the high-frequency E_2g_ first-order mode, respectively. Figure 4b shows the Raman spectra of the Al_3_Mg_2_ alloy decorated with multilayer graphene and clearly shows that compared to the spectra in Figure 4a, the intensity of the D band is much stronger than that of the G band. This can be attributed to the interaction between the Al_3_Mg_2_ alloy and the graphene. The structure of the multilayer graphene was destroyed during the high-energy ball milling and heat treatment process. The intensity ratio of the D band and G band (I_D_/I_G_) in the two samples was also obtained. The I_D_/I_G_ of the multilayer graphene was much lower than that of the Al_3_Mg_2_ alloy decorated with multilayer graphene. The strong D peak suggests that there are more nanocrystalline structures and defects such as distortion, vacancies, and strain on graphitic lattices—which are prevalent features in carbon—in the ball-milled graphene [25]. The Raman spectra that excited at 408.8 and 582.7 cm^−1^ are also displayed in Figure 4b. This can be attributed to the interaction between the multilayer graphene and the Al_3_Mg_2_ alloy [26], which can influence the combustion behavior of the Al_3_Mg_2_ alloy.

### 3.5. SEM Tests on Boron-Decorated and Multilayer Graphene-Decorated Al_3_Mg_2_ Alloys

**Figure 5:**
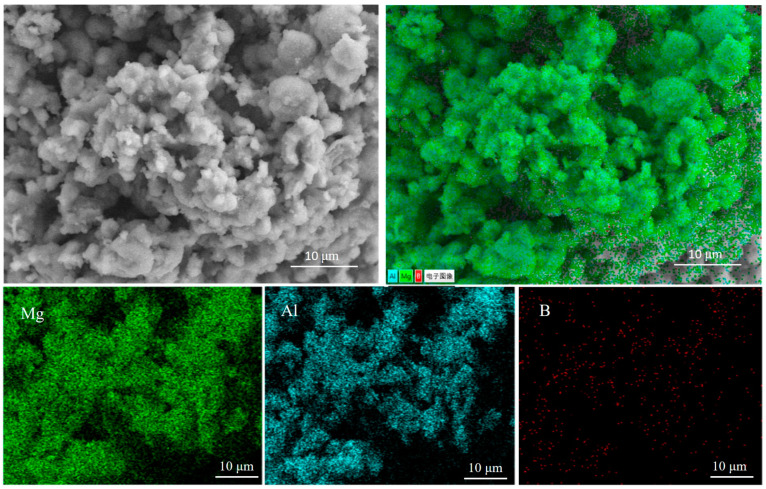
SEM tests on the amorphous boron-decorated Al_3_Mg_2_ alloy.

**Figure 6:**
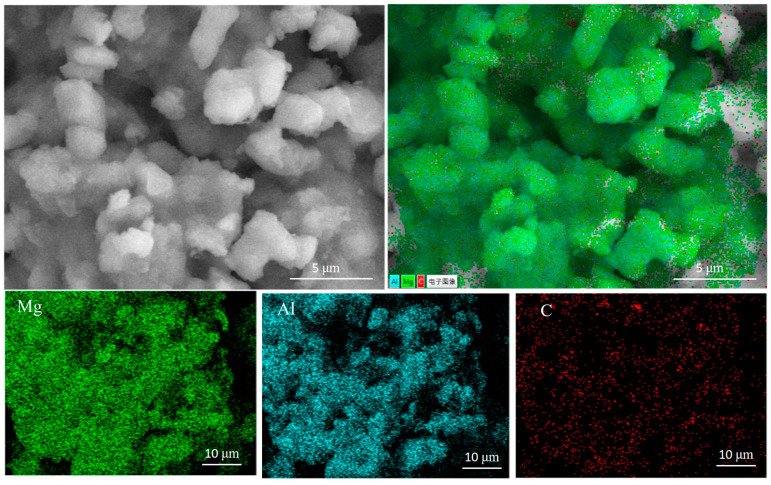
SEM tests on the multilayer graphene-decorated Al_3_Mg_2_ alloy.

The scanning electron microscopy (SEM) test results from the boron-decorated and multilayer graphene-decorated Al_3_Mg_2_ alloys are supplied in Figure 5 and Figure 6, respectively. The corresponding elemental mapping tests are also provided.

Figure 5 reveals that the boron-decorated Al_3_Mg_2_ alloy had an aggregated structure, caused by the high-energy ball milling. All of the Al_3_Mg_2_ alloy particles were ground and the amorphous boron particles were eventually distributed onto the Al_3_Mg_2_ alloy surface. After grinding for a long period of time, all of the boron particles were implanted into the Al_3_Mg_2_ alloy particles. All the elements were evenly distributed. The elemental mapping test results in Figure 5 clearly show that the distribution of the elements was consistent with each other, which means that all of the elements were uniformly distributed by the high-energy ball milling treatment.

The SEM test results from multilayer graphene-decorated Al_3_Mg_2_ alloy are supplied in Figure 6, which also clearly show that the Al_3_Mg_2_ alloy particles were uniformly loaded onto the surface of the multilayer graphene. This can be attributed to the large specific surface area of the multilayer graphene. The amorphous boron particles were evenly distributed onto the layer space of the multilayer graphene after grinding for a certain amount of time. The SEM test results also showed that some particles cohered together, which could be connected by the multilayer graphene. The close contact in the interface between the multilayer graphene and the Al_3_Mg_2_ alloy could promote the combustion characteristics [17].

### 3.6. Combustion Properties of Al_3_Mg_2_, the Boron-Decorated Al_3_Mg_2_ Alloy, and the Multilayer Graphene-Decorated Al_3_Mg_2_ Alloy

The combustion heats of the Al_3_Mg_2_ alloy, B-doped Al_3_Mg_2_ alloy, and multilayer graphene-decorated Al_3_Mg_2_ alloy were tested with the oxygen bomb method. The results showed that the combustion heats of the materials were 27.4 ± 0.20, 32.8 ± 0.18, and 30.5 ± 0.15 MJ/kg, respectively, which means that decoration with both boron and multilayer graphene promoted the combustion of the Al_3_Mg_2_ alloy. Studies on the combustion of the B–Fe system also found that the combustion reactions were altered when boron was doped with iron, which was attributed to the catalytic performance of iron for boron oxidation [27]. Based on this theory, it can be deduced that the combustion of the Al_3_Mg_2_ alloy was catalyzed by the doped boron and the multilayer graphene. It has also been reported that a multiwall carbon nanotube accompanied by Al powder was adopted to improve the combustion performance of hydroxyl ammonium nitrate [28]. The homogeneously dispersed multilayer graphene enhanced the combustion of the Al_3_Mg_2_ alloy. As for the boron-decorated Al_3_Mg_2_ alloy, the combustion of the boron was improved by the high-energy ball milling and the subsequent heat treatment [29,30]. Shabouei et al. developed a multifidelity and multiscale Gaussian process emulator to illustrate the combustion mechanism of combustion at different length scales [31]. It can be concluded that the combustion zone (including both the reaction zone and the diffusion zone) plays an important role in combustion. The combustion performance of Al_3_Mg_2_ alloys were eventually improved with a modified combustion zone width by the decoration of boron and multilayer graphene.

## 4. Conclusions

This paper mainly reported on the structure and combustion heat of Al_3_Mg_2_ alloys decorated with multilayer graphene and boron particles. The results showed that high-energy ball milling can evenly set boron particles into the Al_3_Mg_2_ alloy. The distance between the layers in multilayer graphene can also provide enough space for Al_3_Mg_2_ alloy particles. The even dispersion and appropriate distance between the two phases promoted the combustion of the materials, which eventually resulted in the combustion heat increasing by more than 10 percent.
